# Genetics of Wool and Cashmere Fibre: Progress, Challenges, and Future Research

**DOI:** 10.3390/ani14223228

**Published:** 2024-11-11

**Authors:** Huitong Zhou, Lingrong Bai, Shaobin Li, Wenhao Li, Jiqing Wang, Jinzhong Tao, Jon G. H. Hickford

**Affiliations:** 1 International Wool Research Institute, Faculty of Animal Science and Technology, Gansu Agricultural University, Lanzhou 730070, China; huitong.zhou@lincoln.ac.nz (H.Z.); lingrong.bai@lincolnuni.ac.nz (L.B.); lisb@gsau.edu.cn (S.L.); wangjq@gsau.edu.cn (J.W.); 2 Gene-Marker Laboratory, Faculty of Agriculture and Life Sciences, Lincoln University, Lincoln 7647, New Zealand; 3 Key Laboratory of Animal Genetics and Breeding on Tibetan Plateau, Ministry of Agriculture and Rural Affairs, Qinghai Academy of Animal Science and Veterinary Medicine, Qinghai University, Xining 810016, China; qhdxlwh@163.com; 4 School of Animal Science and Technology, Ningxia University, Yinchuan 750021, China; tao_jz@nxu.edu.cn

**Keywords:** keratin, keratin-associated protein, variation, genetic association, haplotype, omics, wool traits

## Abstract

Wool (sheep) and cashmere (goat) fibres have unique biological, physical, and chemical properties and these fibres are becoming more important as the demand for natural products increases. However, these complex protein fibres are at times compromised by natural variability in their properties, and this can impact their use and value. Genetic improvement via selection and breeding can partly overcome this problem, enabling the farming of sheep and goats that produce more desirable fibre. This review explores the challenges in improving wool and cashmere fibre characteristics using genetics, with a focus on improving our understanding of the key protein components of fibres, wool keratins and keratin-associated proteins (KAPs). Despite progress in our knowledge of these proteins, gaining a better understanding of them and how they affect these fibres remains an ongoing challenge. This is not straight-forward, given the large number of similar yet unique genes that produce the proteins and the gaps that remain in their identification and characterisation. More research is required to clarify gene and protein sequence variability and the location and patterns of gene expression, which in turn limits our understanding of fibre growth and variation. Several aspects that currently hinder our progress in this quest include the incomplete identification of all the genes and weaknesses in the approaches used to characterise them, including newer omics technologies. We describe future research directions and challenges, including the need for ongoing gene identification, variation characterisation, and gene expression analysis and association studies to enable further improvement to these valuable natural fibres.

## Simple Summary

Wool and cashmere are highly valued for their natural properties and environmental benefits. However, their quality can vary due to natural differences in fibres, affecting their usefulness and value. This review examines how selecting and breeding animals for better fibres can address these issues. It focuses on the challenge of understanding the proteins in wool and cashmere, which are crucial for their quality. Despite advances, identifying and characterising the many genes involved remains difficult. Continued research is needed to improve our knowledge of these genes and proteins, which will help enhance the quality of wool and cashmere products and make them even more valuable in various industries.

## 1. Introduction

Wool and cashmere fibres have long been valued for their properties and are prized by many industries, from the manufacture of interior textiles and carpets to insulation and filters, as well as inner and outer layers of clothing. With a growing global awareness of health, well-being, environmental issues, and sustainability, there is a renewed interest in these fibres. Despite this, natural variation in the fibres can impede their uses and economic value.

Recognising the importance of wool and cashmere fibres and the need for improvement in fibre uniformity, genetic enhancement through the selection and breeding of sheep and goats with better fibre properties remains important. Central to this approach is the need to have a comprehensive understanding of the genetic mechanisms governing fibre attributes, with a key focus being the genes that encode the main protein components of the fibres: wool keratins and keratin-associated proteins (KAPs). Despite considerable effort, our understanding of these genes and proteins is still incomplete, with gaps in the identification of genes, the characterisation of genetic variation, our understanding of gene expression, and the impact of these things on fibre traits. These limitations hinder our progress towards genetic improvement.

## 2. The Heritability of Key Wool and Cashmere Fibre Traits

Wool and cashmere fibres are not uniform. They are affected by genetic, environmental, and management factors. The traditional method of predicting the value of wool and cashmere is visual assessment and ‘handle’, or how the fibre feels, but this is a subjective method and is accordingly inconsistent and inaccurate.

Use of specialised instruments makes the measurement of wool and cashmere more accurate. This is beneficial not only to the grower but also to the buyer and the processor. Growers can use test results to improve breeding programmes and optimise farm management practices, while buyers and processors will obtain an indication of the true value of sale lots and how they might subsequently perform in processing.

The major factor or characteristic affecting the value of wool and cashmere is mean fibre diameter (MFD) [1,2]. Merino wool is generally considered as the finest wool, with fibre diameters typically ranging from less than 12 µm to 24 µm. Mean fibre diameter is also arguably the most important trait for cashmere producers, as textile definitions usually consider fibres 19 μm or less to be cashmere.

The multitude of effects of different fibre characteristics on fibre use are too numerous to detail in this review. However, many of the key determinants of fibre quality are highly heritable, suggesting they are under genetic control and can be bred for. Heritability estimates for some of the key wool and cashmere traits are listed in Table 1 and Table 2, respectively.

## 3. Potential for the Further Improvement of Wool and Cashmere Fibres

Wool and cashmere fibres are highly valued for their unique properties, yet their market remains limited but holds potential for growth. To capitalise on this potential, it is essential to enhance the quality of wool and cashmere fibres to meet diverse application standards. Given the heritability of fibre traits, genetic improvement through selective breeding offers a promising path to improve fibre production, supporting market growth and broader use in various industries.

### 3.1. Market Trends and Growth Prospects

Wool and cashmere fibre production represents a modest fraction (approximately 1%) of the global textile fibre supply [22]. Future demand for these fibres will largely rely on their ability to grow existing markets and become part of new products, but challenges, such as the currently higher production and processing costs compared to synthetic and other natural fibres like cotton, must be addressed [23]. It has been argued, given the current difference in production and processing cost, that marketing strategies should position wool and cashmere as luxury niche products, targeting rising middle-class consumers in Asia [22]. However, if the cost of product disposal and carbon and water footprint is fully factored into product cost, then alternative fibres like synthetics and cotton may become more expensive than wool to use in products.

Emerging opportunities in sectors like next-to-skin knit-wear and leisure-wear present further avenues for growth in wool and cashmere use [24], but these clothing markets require fibres with a low diameter (less than 18 μm) for use as base layers against the skin [22]. Increasing public awareness of the properties of these fibres, coupled with the global trends towards healthy and eco-friendly products, will likely shape future demand, especially as the biodegradability and renewability of wool and cashmere fibres [25] should position them as superior alternatives to synthetic fibres. So-called ‘eco-positioning’ may further enhance their appeal to environmentally conscious consumers.

Wool has also recently found applications as an alternative material in some industrial sectors [26]. The growing demand for alternative materials, particularly in building, has driven the production of wool fibres with unique properties, such as with improved thermal and sound insulation capabilities [25]. Wool with enlarged pores is now commercially available and utilised for absorbing dyes, cleaning up oil spills, and capturing volatile organic compounds, with this contributing to more environmentally friendly practices [27,28,29,30]. Wool is naturally flame-retardant and does not ignite easily. It burns with a self-extinguishing flame and forms a soft ash residue, unlike synthetic fibres, which form a hard, molten bead residue that has melt–drip behaviour [31]. Wool’s unique composition also makes it a choice for upholstery in light aircraft, ships, and trains [32]. Its inherent self-extinguishing property if exposed to flames makes wool a promising material for heat-generating appliances [32], further broadening its range of industrial applications.

The anticipated growth in demand for these fibres will be supported by advancements in sheep and cashmere goat farming practices and genetic improvements that enhance yield and quality. However, challenges such as variability in fibre traits impact the use of wool. Traits like fibre diameter, staple length, and crimp consistency can vary greatly between individual animals and breeds, with this affecting both fibre quality and their subsequent usability. Obtaining greater consistency in key traits is crucial if wool and cashmere are to meet the high standards needed for various applications from fashion to industrial uses.

### 3.2. Breeding for Better Wool and Cashmere

Research indicates that wool and cashmere traits are moderately to highly heritable (See Table 1 and Table 2, respectively). This suggests that these traits are under genetic control and can thus be improved through selective breeding approaches. In countries with large wool industries such as Australia and New Zealand, farmer-led organisations like Sheep GENETICS (Meat & Livestock Australia, Armidale, NSW, Australia) and B + LNZ Genetics (Beef + Lamb New Zealand Incorporated, Wellington, New Zealand), respectively, provide breeding tools including estimated breeding values for selected key wool traits.

The moderate to high heritability of key wool and cashmere traits offers a promising avenue for genetic enhancement to address fibre variability issues. A better understanding of the genetic factors that influence specific traits would further enable sheep and goat breeders to develop targeted breeding approaches to enhance desirable qualities while minimising undesirable fibre variation. Genetic improvement would not only foster the production of superior quality fibres but also potentially enhance the efficiency and sustainability of production. Accordingly, as our understanding of the genetic basis of fibre traits deepens, the potential for substantial advancements in fibre quality and production efficiency would increase, underpinning a brighter future for these fibres.

## 4. The Genetics of Wool and Cashmere Fibre Traits

The heritability estimates for wool and cashmere fibre traits suggest a major genetic influence and possibly suggest relatively simple genetic control. However, studies using both candidate gene associations and omics approaches have revealed numerous genes associated with these traits. This suggests that the genetic basis of wool and cashmere traits may be more complex than presumed. The evidence on hand suggests the involvement of many genes of small effect or of yet-to-be-identified genes of major effect that GWAS and RNA-Seq approaches have failed to resolve.

The known genes associated with key fibre traits are listed below, and they are categorised based on whether their effect was ascertained by candidate gene association studies or by omics studies.

### 4.1. Genes Associated with Wool and Cashmere Traits Identified by Candidate Gene Approaches

Wool fibres are primarily composed of proteins, accounting for nearly all the dry wool fibre mass [33]. These proteins include wool keratins and KAPs. The structure of a protein determines its function, so the structures of individual wool proteins and their relatively proportions in the fibre are believed to influence fibre structure and properties.

Wool keratins are classified as either type I or type II proteins and form heterodimers that assemble into keratin intermediate filaments (KIFs), the main structural component of wool fibres [34]. KAPs are classified into three groups: high-sulphur (HS), ultrahigh-sulphur (UHS), and high-glycine–tyrosine (HGT) proteins. They form a complex matrix that crosslinks and embeds KIFs [34].

Understanding the genes encoding KAPs and wool keratins is probably crucial to understanding the genetic factors that determine the characteristics of wool fibres, so these genes (*KRTAPs* and *KRTs,* respectively) represent key targets for candidate gene association studies.

In sheep, 21 *KRTAPs* and 10 wool *KRTs* have been reported to be associated with wool traits to date (Table 3), while in goats, 13 *KRTAPs* have been linked to cashmere fibre traits (Table 4). These findings reinforce the idea that *KRTAPs* and *KRTs* regulate wool fibre characteristics. Considering the number of genes involved and the potential for the discovery and characterisation of more genes, individual gene effects are expected to be minor, and thus major gene effects seem unlikely.

The associations detected also appear to reflect the activity of individual genes, rather than linkage to nearby genes or loci. This is because different genetic associations are observed with genes that are positioned very close to each other on the same chromosome. What is more, inconsistencies in the associations when comparing different breeds/types of sheep and goats possibly reflects gene–environment (G × E) interactions. Together, this suggests a further need for robust studies involving more animals from a diversity of breeds.

Among proteins in wool fibre that are neither KAPs nor wool keratins, trichohyalin (TCHH) is a large α-helix-rich insoluble protein that is abundant in the inner root sheath (approximately one-third of total protein) and wool’s medulla [88]. It forms part of the interfilamentous matrix, cross-linking to itself and to the KIFs. This stabilises the links between the keratin filaments and the cell envelope. This cross-linking provides mechanical strength in the mature fibre structure [89]. Variation in the ovine TCHH gene has been associated with mean fibre curvature (MFC; [90]).

In humans, variation in the TCHH gene has been linked to hair curliness in Europeans [91], perhaps reflecting its association with MFC in sheep. A meta-analysis across diverse populations identified an association between *TCHH* and hair shape [92], and mutations in human *KRT81*, *KRT83* and *KRT86* cause Monilethrix, a condition characterised by the presence of abnormal hair shafts [93,94,95].

Together, these findings about KAPs, keratins, and TCHH highlight the conserved functional significance of these proteins across species and support the role of these proteins (and thus their genes) in determining fibre properties. The potential for proteomics to be used to improve wool traits has been reviewed [96] and the protein differences between breeds are discussed in studies like that of Plowman et al. [97].

### 4.2. Genes Associated with Wool and Cashmere Traits by Omics Analyses

Recent advances in omics technologies have led to effort to use these methods to identify genes that regulate wool and cashmere characteristics. There appear to be two main typing approaches used: genome-wide association studies (GWAS), including SNP chip typing, and transcriptome analyses, which compare gene expression profiles. These approaches face specific challenges, and they have provided varying and as yet inconsistent insights into fibre properties.

#### 4.2.1. Genes Identified by GWAS

Efforts have been made to identify genes associated with wool traits using GWAS in sheep, but with only limited research undertaken in goats. In these studies, it is notable that the detection of associations with wool protein genes has been minimal, or absent, while most of the genes identified to date have no functions that are confirmed to be related to wool traits.

For example, Arzik et al. [98] analysed 426 Akkaraman sheep using the Axiom 50K Ovine Genotyping Array and identified several genes and genomic regions associated with wool fibre and fleece characteristics. These included links between *TRIM2*, *MND1*, *TLR2*, *RNF175,* and two undefined loci (*LOC101122892* and *LOC106991694*) on chromosome 17 and fibre diameter; *CEP290* and *TMTC3* on chromosome 3 and fibre diameter; *RERE*, *SLC45A1*, *LOC101118971*, and *LOC105609137* on chromosome 12 and staple length; and *MORN1*, *SKI*, *FAAP20*, *PRKCZ*, *GABRD*, *CFAP74*, *CALML6*, *TMEM52*, *LOC106991467*, *LOC106991455*, *LOC105616534*, and *LOC105609719* on chromosome 12 and yearling staple length.

Becker et al. [99] used the same SNP array to conduct a GWAS on United States Rambouillet sheep. They also identified several associations, including links between a marker in the ribosomal protein L17-like (*LOC121818710*) on chromosome 1 and average fibre diameter, between a marker in the intron of the ATP binding cassette sub-family C member 8 gene (*ABCC8*) on chromosome 15 and skin wrinkle score, and between the intron of the unc-51 like kinase 4 gene (*ULK4*) gene on chromosome 19 and face wool score. There were five other associations with markers on chromosomes 1 (2× independent markers), 2, 4 and 15, and wool average fibre diameter, clean fleece weight, face wool score, and staple length, respectively. At most, these markers explain 8.25% of the proportion of variance explained for the trait, so, arguably, they are of minor effect.

Wang et al. [100] analysed 765 Chinese Merino sheep of the JunKen type using Illumina 50K SNP chips and identified 28 genome-wide significant SNPs associated with various wool traits, of which 12 were near genes *YWHAZ*, *KRTCAP3*, *TSPEAR*, *PIK3R4*, *KIF16B*, *PTPN3*, *GPRC5A*, *DDX47*, *TCF9*, *TPTE2*, *EPHA5*, and *NBEA*. The markers spanned ovine chromosomes 1, 2, 3, 4, 6, 8, 9, 10, 11, 13, 23, and 25 and were associated with traits including fibre diameter, coefficient of variation of fibre diameter, fineness dispersion, and crimp.

It is notable that, despite the use of similar technologies, there is no obvious cross-over in the genes identified in these three studies.

Zhao et al. [101] re-sequenced 460 sheep from four Chinese fine-wool breeds and detected 57 genome-wide SNPs and 30 genes associated with various wool traits, but none of these genes encoded keratins or KAPs. In a separate GWAS resequencing analysis by the same research group [102], using data from 577 sheep of the same breeds, 16 SNPs were identified at the genome-wide level and 79 SNPs above the suggestive significant threshold, with the authors reporting a total of 66 genes associated with various yearling wool traits. Only one gene (*KRTAP6-1*) encoded a wool protein. The study did not refer to the earlier study, despite it being published not even five months earlier, and no effort was made to reconcile the genes and markers described in both studies. Rather surprisingly, despite the similarity of the sheep studied, albeit with a slight difference in their age (over 550 days in the first study and 14 ± 1 months in the second study), nearly all the SNPs and genes identified were different.

In goats, Wang et al. [103] studied Inner Mongolia cashmere goats using Illumina GoatSNP52K Beadchips and identified four SNPs at genome-wide significant levels and genes like *FGF12*, *SEMA3D*, *EVOL*, and *SOX5* associated with cashmere traits. None of these genes were revealed in the sheep studies described above, despite the genetic similarity of sheep and goats.

A more recent study [104] has suggested that genomic selection might be used to improve wool traits, but accuracies for the selection for key traits tended to be low. Traits with a high heritability and a large training population did tend to result in higher accuracies than those with an average heritability across populations. This study again listed multiple candidate genes that were associated with variation in wool or hair growth but did not find any significant SNPs within the genes described for the Chinese Merino sheep studied by Wang et al. [100], despite studying Merino and Merino-cross sheep.

It is notable that there is little to no concordance between the separate GWAS for wool traits or any consistency with the studies of the individual *KRTAPs*, *KRTs*, and *TCHH*. Is it because different sheep in different populations are being studied at different times of their lives, or are other factors, such as phenotypic variability, masking the underlying genetics of the wool traits? While the latter argument is a possibility, it is weakened by the weight of evidence suggesting that many key wool traits, such as MFD, are highly heritable [104].

#### 4.2.2. Challenges with Using SNP Chip Typing Approaches

The rare detection of *KRTs*, *KRTAPs*, or *TCHH* using the SNP chip approaches might, however, be attributable to the low density of SNPs on these chips. Most *KRTAPs* identified to date exhibit much higher SNP densities than the average density of 4.9 SNPs per kb suggested across the sheep genome [105]. The Illumina Ovine SNP50K BeadChip, with an average distance of 50.9 kb between markers, yields a density of approximately 0.0196 SNPs per kb of DNA sequence, and the so-called ‘high-density’ Illumina Ovine Infinium HD SNP BeadChip (600 K), the highest-density ovine SNP chip available, also likely has a SNP density that is insufficient to adequately describe the known variation in wool *KRTs* and *KRTAPs*.

To address this issue, one may then ask whether specialised SNP chips could or should be designed to target the variation in wool keratin and KAP genes. This might be feasible, but it needs to be noted that the variation in wool keratin genes has not been well characterised overall, and that our current understanding of the KAP genes identified and annotated to date suggests a sizeable challenge in trying to use SNP chips to better understand the structure and function of these genes.

**Figure 1:**
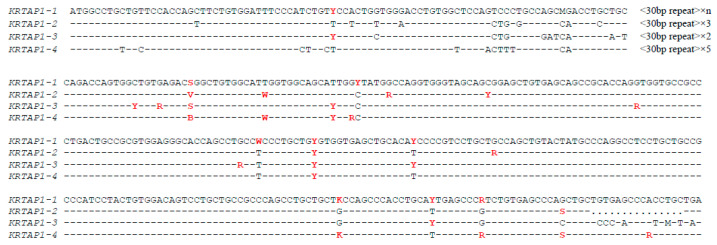
Sequence alignment of the ovine KAP1-n genes showing extensive nucleotide sequence conservation at and around SNP positions. Degenerate symbols represent nucleotide sequences at each SNP: R = A/G; Y = C/T; S = C/G; K = G/T; M = A/C; W = A/T; B = C/G/T; V = A/C/G, W = A/T. SNPs with their nucleotide sequences and flanking sequences shared by family members are highlighted in red and bolded. Nucleotide sequences identical to the top sequence are indicated by dashes.

To illustrate this point, with the KAP genes characterised to date, it has been revealed that certain SNP sequences along with their surrounding sequences are not unique to specific genes. They are instead shared across multiple members within a gene family [106,107]. This is exemplified by the ovine KAP1 genes (Figure 1), where some SNPs are shared across four genes, but with conserved sequences around them. Similar observations have also been made with other KAP families that contain multiple members. This lack of specificity could lead to misinterpretation of signals and subsequently mis-typing of the genes. The challenge in even something as simple as designing specific oligonucleotide probes for these SNPs raises doubts about the suitability of SNP chip typing technologies for wool and cashmere fibre research.

Equally, the low detection of keratin and KAP genes using re-sequencing approaches suggests that there are other complicating factors in undertaking association studies. One of these may be that, for any given trait, variation is underpinned by the involvement of numerous genes, each exerting a small effect. This would make the individual genetic contributions to the trait difficult to detect.

Another factor may be that it is extended SNP haplotypes, rather than individual SNPs, that play a role in determining phenotype. The wool keratin and KAP genes typically contain multiple SNPs. This results in extended SNP haplotypes that can also be referred to as ‘variants’ or alleles in both sheep [74,108,109,110] and goats [65,111,112]. Association studies solely based on individual SNP typing may therefore oversimplify the genetic challenge. This is because comparing bi-nucleotide variants at each SNP is equivalent to combining all SNP haplotypes into two groups and comparing them, with this potentially masking genuine associations, especially when it is known that some KAP genes have more than two alleles. Consequently, this approach may fail to detect associations.

In this context, while GWAS have proven successful for detecting simple mutations or genetic variation associated with qualitative traits, such as describing the gene mutations causing microphthalmia in sheep [113], the slick-haired condition in cattle [114], or the presence or absence of horns in cattle [115], they may struggle with genetic complexity, as is found with the wool keratin and KAP genes. Overall, while GWAS can be effective in identifying genes with major effects on quantitative traits, such as prolificacy [116], for some quantitative traits like wool traits, which might be influenced by a multitude of genes and the environment, GWAS approaches may prove less suitable.

The limitations of SNP chip technology in detecting variation in keratin and KAP genes, and how that may affect fibre traits, emphasise the need for alternative approaches. While GWAS have proven valuable for certain traits, their effectiveness for quantitative traits like wool remains uncertain. Accordingly, some commentators have concluded that the initial anticipation of discovering genes with major effects on production traits in livestock genetics has largely fallen short because of the predominance of traits where numerous genes with minor additive effects affect major production parameters [23].

#### 4.2.3. Genes Identified by Transcriptome Analyses

Studies using RNA-Seq to identify differentially expressed genes (DEGs) associated with wool and cashmere fibre traits have also been undertaken. However, like the genes identified by GWAS, only a few wool keratin and KAP genes have been identified in these studies, and many of the genes identified have no known role in regulating fibre traits.

For example, Ma et al. [117] conducted transcriptome analysis to find genes associated with wool fineness in skin tissues of Subo Merino (superfine-wool) and Chinese Merino (fine-wool) sheep. They identified 16 DEGs associated with wool fineness, including *CACNA1S*, *GP5*, *HSF5*, *SLITRK2*, *CREB3L4*, *COL1A1*, *PTPRR*, *SFRP4*, *COL6A6*, *COL6A5*, *LAMA1*, and others. In contrast, Zhang et al. [118] compared the skin gene expression profiles of fine-wool Super Merino and coarse-wool Small Tail Han sheep and identified 435 DEGs, with 7 of them being *KRTs* (*KRT36* and *KRT79*) or *KRTAPs* (*KRTAP1-1*, *KRTAP4-9*, *KRTAP6-1*, *KRTAP6-2L,* and *KRTAP9-2*). Wang et al. [119] analysed skin RNA profiles from three pairs of modern fine and ancestral-like coarse-wool-breed lambs and identified 728 up-regulated and 805 down-regulated genes in the skin of ancestral-like coarse-wool-breed lambs, compared to modern fine lambs. Among these, only five were *KRTs* (*KRT17*, *KRT25*, *KRT27,* and *KRT71*) or *KRTAPs* (*KRTAP5-4*). Qin et al. [120] used RNA-Seq to analyse Liaoning cashmere goat skin from fine and coarse wool samples and identified a total of 427 DEGs, with no caprine keratin and KAP genes being identified. Recently, Jin et al. [121] suggested the melatonin-responsive lncRNA018392 accelerates the cell cycle and may be implicated in cashmere growth, and Wang et al. [122] reported that *CXCL8* may regulate cashmere fineness.

#### 4.2.4. Challenges Posed by Incomplete Gene Reference Databases in RNA-Seq Approaches

The somewhat rare identification of keratin and KAP genes using RNA-Seq is possibly because of the relatively small number of these genes available in the reference databases that are used to identify DEGs. The DEGs detected with RNA-Seq approaches may also be influenced by the variability observed between samples within the same groups and compounded by error from the small sample sizes often used in these analyses. That is, many of the RNA-Seq studies compare small groups of samples (usually around three per group) and rely on mean data from within these groups for comparisons.

However, given the sizeable variation in individual expression profiles within these small groups, comparisons based solely on the mean may not accurately reflect the true phenotypic or genotypic differences between groups. Care is therefore suggested when interpreting RNA-Seq results obtained from small-sample-size studies, and increased sample sizes may be necessary to obtain more robust, repeatable, and reliable findings.

In conclusion, despite advancements, the candidate gene approach has primarily focused on a small subset of known wool protein genes and the associations revealed between those genes and fibre traits need to be validated across more sheep breeds and larger sample sizes. On the other hand, omics approaches encounter challenges such as the sparse annotation of many genes, let alone wool protein genes, the presence of non-specific SNPs shared across genes, and inadequate SNP density on the commonly used chips.

The importance of compiling extended SNP haplotypes as opposed to studying individual SNPs, and the multiallelic variation in many of the KAP and wool keratin genes, which may influence protein structure and/or expression, will further complicate omics studies. Taken together, these factors highlight the need for a more comprehensive characterisation of wool protein genes, the development of improved methods, and more genetic association studies to address the current limitations of understanding. These challenges are further discussed below.

## 5. Current Issues in Genetic Improvement

Current efforts to enhance wool and cashmere fibre traits are hindered by the limited understanding of the genes that regulate these characteristics. Despite advancements in genetics research and the availability of genome sequencing, the complexity of wool keratin and KAP genes, any other genes that may be affecting fibre traits, and their expression patterns are poorly understood.

This knowledge gap hampers the use of marker-assisted breeding approaches and thus limits improvement in fibre quality and production efficiency. Addressing this gap requires further research endeavour and the development of improved analytical approaches.

### 5.1. Limited Knowledge of KAP and Keratin Genes

Despite the first research on wool keratin and KAPs in sheep beginning nearly five decades ago, progress in identifying, annotating, and characterising these genes and proteins has been much slower than that made in humans. Human research has identified what is presumed to be the complete catalogue of all hair keratin and KAP genes, comprising 17 hair keratin genes [11 type I (*KRT31*, *KRT32*, *KRT33A*, *KRT33B*, and *KRT34-KRT40*) and 6 type II (*KRT81-KRT86*)] [123] and 89 KAP genes (23 HS-KAP, 46 UHS-KAP, and 20 HGT-KAP genes) [124,125,126].

In sheep, a comparable number of wool keratin genes have been identified, with 10 type I genes (*KRT31*, *KRT32*, *KRT33A*, *KRT33B*, and *KRT34-KRT40,* excluding *KRT37*) and 7 type II genes (*KRT81-KRT87*) being documented [127,128,129]. The type I keratin gene *KRT37* has not been identified in sheep; instead, there is an additional type II keratin gene, *KRT87*. While the exact number of wool keratin genes in goats remains undefined, our analysis of the goat genome assembly sequence (ARS1, RefSeq GCF_001704415.2; formerly ASM170441v1) suggests the presence of 10 type I and 7 type II genes, which is consistent with what has been found in sheep.

As for KAP genes, by searching the whole sequence for ‘signatures’ that would suggest a KAP gene, our analysis of the sheep genome assembly sequence (ARS-UI_Ramb_v2.0; RefSeq GCA_016772045.1) reveals the potential presence of 102 KAP genes, consisting of 23 HS-KAP, 48 UHS-KAP, and 31 HGT-KAP genes. The signature for a KAP gene is not solely based on its putative amino acid composition upon translation (e.g., whether they are cysteine-rich or HGT-rich), but also on its small size, lack of introns, chromosomal clustering, DNA sequence similarities with other known KAP genes, and unique DNA sequence patterns, such as the occurrence of typically in-frame tandem repeats. To date, the number of ovine KAP genes identified and described in any detail is 32, including those documented in Zhou et al. [107] and the recently identified genes *KRTAP36-2* [67] and *KRTAP19-5* [58]. This represents approximately one-third of the potential total count.

Similarly, our analysis of the goat genome assembly sequence (ARS1, RefSeq GCF_001704415.2; formerly ASM170441v1), conducted using the same approach of searching for signatures that one might expect for KAP genes, suggests the presence of 99 KAP genes, encompassing 23 HS-KAPs, 45 UHS-KAP, and 31 HGT-KAP genes. However, the count of KAP genes investigated in any detail in goats is very limited, with only 23 KAP genes reported so far, including those listed in Zhou et al. [107] and the recently identified genes *KRTAP6-2* [79], *KRTAP6-5* [78], and *KRTAP22-2* [77]. This accounts for just one quarter of the anticipated total.

This highlights the limited representation of KAP genes in the current reference sequence databases, and this shortfall likely hinders our understanding of the genetic basis of wool and cashmere traits, as most of the sheep and goat KAPs remain unannotated, despite their sequences potentially being present in the genome assemblies. This means that their coding sequences have not been formally identified and reported, which poses a major limitation to omics studies that rely on these reference sequences to identify genes. The ongoing characterisation of these un-annotated KAP genes is therefore crucial.

It could therefore be argued that this is a priority before too many more omics studies are undertaken, as these studies are likely to be compromised by the missing details about KAPs. The characterisation of these genes would ensure better outcomes and mitigate the risk of data misinterpretation or the failure to detect important associations. Neglecting this issue not only risks producing misleading results or uninformative results but also wastes resources, including time and research funding. We would therefore recommend far greater effort is made forthwith to fully catalogue the KAP genes, especially if we are to advance our understanding of wool and cashmere traits, optimise genetic improvement strategies, and ultimately obtain better fibre.

### 5.2. Inadequate Understanding of Wool Protein Gene Expression Patterns

Some research on the spatial and sequential expression patterns of wool keratin genes in sheep has been conducted, but information on goats remains limited.

**Figure 2:**
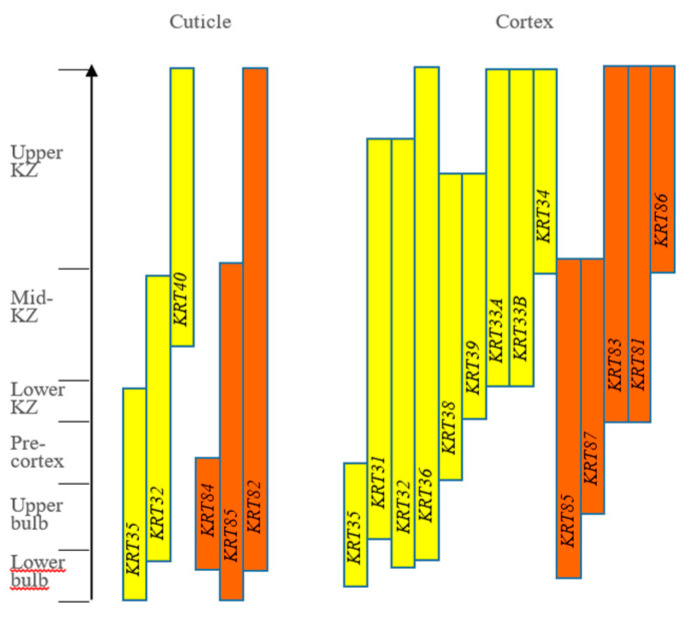
Schematic presentation of expression zone and expression sequential of ovine *KRT* genes in wool follicles. Proximal follicle zones are labelled on the left. The arrow indicates direction of sequential gene expression. Type I and type II genes are colour-coded in yellow and orange, respectively. KZ strands for the keratogenous zone. This figure is adapted from Yu et al. [130].

In sheep, in situ mRNA analysis has shown that *KRT40*, *KRT82*, and *KRT84* are exclusively expressed in the fibre cuticle, while *KRT32*, *KRT35*, and *KRT85* are expressed in both the cuticle and the cortex. The remaining 11 genes (*KRT31*, *KRT33A*, *KRT33B*, *KRT34*, *KRT36*, *KRT38-39*, *KRT81*, *KRT83,* and *KRT86-87*) are solely expressed in the cortex (Figure 2; [130]).

In both the cuticle and cortex, *KRT35* and *KRT85* expression precedes *KRT32*, *KRT82,* and *KRT84* expression in the cuticle. While *KRT82* expression extends to the upper keratogenous zone, *KRT84* mRNA is restricted to the follicle bulb. The expression of *KRT40* is confined to the fibre cuticle in the mid-to-upper keratogenous zone [130]. In the cortex, the expression of *KRT35* and *KRT85* is followed by *KRT32*, *KRT36*, *KRT31*, *KRT38,* and *KRT87*. The expression of *KRT33A*, *KRT33B*, *KRT81,* and *KRT83* occurs in the mid-to-upper keratogenous zone, with *KRT34* and *KRT86* expression occurring even further distally in the upper keratogenous zone [130]. Several keratin genes, including *KRT31-34*, *KRT36*, *KRT38*, *KRT39*, *KRT81*, *KRT83*, *KRT84*, *KRT86*, and *KRT87*, are detected in the medulla, with *KRT34* and *KRT36* having high levels of expression [130].

The expression patterns of wool keratin genes are similar to those observed for the human hair keratin genes [131,132], but some differences exist. A notable difference is that there are more wool keratin genes from both type I and type II groups expressed from the lower region to the upper region of the wool follicle cortex. Unlike the human hair follicle, *KRT84* is expressed in the wool follicle, alongside the expression of a new type II keratin gene, *KRT87* [130]. This possibly increases the likelihood of type I and type II keratin pairs forming in the cortex, with this likely adding increased complexity to the wool fibre structure.

Research on the spatial and sequential expression patterns of *KRTAPs* in sheep has been limited to the small number of KAP families identified three decades ago. Following the onset of wool *KRT* expression in bulb and cortical cells, ovine *KRTAPs* exhibit sequential expression patterns in the lower to mid-follicle shaft. The *KRTAPs* of the KAP6, KAP7, and KAP8 families are the first to be expressed, predominantly in cells of orthocortical lineage [34]. Subsequently, the expression of *KRTAPs* from the KAP1, KAP2, and KAP3 families occurs in cortical cells, initially in the region of the cortex complementary to that expressing the KAP6, KAP7, and KAP8 families, but eventually extends over time to all cortical cells [34]. A gene from the KAP4 family is expressed slightly later, primarily within paracortical cells [133]. Genes from the KAP5 family and a KAP10 gene (for which sequence information is not available on public databases) are expressed late in wool cuticle differentiation [34,134,135].

Given that information about the expression patterns of *KRTAPs* is relatively sparse and that little is known about many other *KRTAPs* in sheep, as well as given the complete lack of information on *KRTAP* expression in goats, it is difficult to obtain a generalised idea or overview of KAP expression patterns.

### 5.3. Reduced Focus on Individual Wool Protein Genes in Favour of Omics Studies

Wool keratins and KAPs are the main structural components of wool and cashmere fibres. Consequently, genes encoding these proteins are a primary target of candidate gene association studies. Despite their importance, limited effort has been made of late to characterise these genes more fully in sheep and goats and to investigate their effect on wool and cashmere traits. Consequently, many KAP genes remains unidentified in sheep and goats.

In contrast, there is an increasing reliance on omics-based approaches to understand wool and cashmere fibre characteristics, including genomics (genes), transcriptomics (messenger RNA), and proteomics (proteins). Advances in laboratory protocols, data storage, and bioinformatics have made it possible to generate vast amounts of omics data, and while omics technologies offer powerful tools to dissect the phenotypic and functional network of genes and proteins, they face significant criticisms and challenges too. Issues such as reproducibility, noise from background hybridisation signals, and false detections, as well as the exploratory nature of omics approaches, can lead to scepticism about the approach and the utility of findings.

Other researchers have noted that omics findings often cannot be replicated [136,137] and the experiments are generally complex and require careful design to minimise spurious variation and account for biological and technical parameters that influence the results [138]. Noise from the large volume of data generated can overwhelm meaningful signals, potentially explaining why few relevant candidate genes are detected [136,138].

The exploratory nature of omics research is also often touted as being a ‘fishing expedition’ without clear targets, where researchers blindly hope to find something interesting but often fall short. These issues are inter-related as reproducibility problems arise from having ‘noisy’ data [139], and intra- and inter-experimental quality can vary.

While we now have an unprecedented capability to collect molecular-level data and the computational power to store and analyse it, our understanding of these data appears to have lagged far behind its accumulation. This has led to a degree of fragmentation in the science that only specialists can navigate. This stands in stark contrast with the past era of molecular genetics, where significant progress was made through the design of more thorough experiments using simpler and arguably more readily controlled approaches. In this respect, it has been said that some scientists are now over-enthusiastic about using ‘high-tech’ omics for its own sake, falling into a pattern of ‘low input, high throughput, no output’ research [140]. However, this type of research appears to dominate current scientific journals and thinking, but at times it arguably contributes little to meaningful scientific advancement.

In addition to these general issues, the unique characteristics of wool keratin and KAP proteins and their genes pose further challenges to omics approaches. Wool keratins and KAPs typically share high degrees of sequence similarity within their types or families [34,141]. This similarity can lead to an increased rate of multi-mapping fragments, where a fragment overlaps more than one protein or gene, with this resulting in assignment ambiguity [142]. Disregarding multi-mapping sequences will lead to biases in biological and functional assessments [143]. This issue may be more severe in proteomics, where the optimal peptide length for detection is usually short (8–15 amino acids).

**Figure 3:**
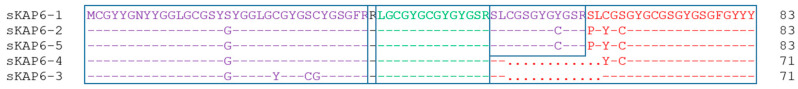
Predicted peptides generated for five sheep KAP6 family members cleaved by trypsin. Individual peptides from each family member protein are illustrated in different colour and boxed. Amino acids identical to the top sequences are represented by dashes, and dots are introduced to improve sequence alignment. The length of each protein is indicated at the end of the sequence.

In this respect, the analysis of short peptides might be very problematic for wool keratins and KAPs because of the high similarity of the protein sequences. As an example of this, the tryptic digestion of proteins from the five members of the ovine KAP6 family is predicted to generate some peptides that are identical or highly similar to other family members (Figure 3), and this would compromise protein annotation.

Equally, wool protein genes are characteristically polymorphic, and many of the genes possess multiple variant sequences [66,107], with up to 11 variants identified for *KRTAP1-2* [70,144]. The flexibility or stringency of the mismatch policy employed in analysing these genes is thus crucially important to accurately mapping reads, given that reads containing one or more SNPs have a reduced likelihood of being successfully mapped [145]. Furthermore, genetic variation in KAP genes includes length variations, such as short-nucleotide insertions or deletions (indels), in both sheep and goat genes [107]. This further complicates the annotation of the proteins and genes. Algorithms that lack effective gapped-alignment capabilities often struggle to align reads containing indels [146].

As wool keratins and KAPs are the primary structural elements in wool and cashmere fibres, it is anticipated that these proteins would be abundant in these fibres and that their genes would exhibit high levels of expression in the fibre follicle. It is therefore unlikely that there will be substantial variation in the quantity of these proteins or the level of expression of the encoding genes, and it might also be anticipated that even subtle changes in expression might influence wool and cashmere fibre traits.

In contrast, proteins that are rare or present in low quantities, and genes that exhibit weak expression may have fold-level changes in expression due to their small quantity. This may partially explain why many proteins or genes identified as differentially expressed in wool and cashmere fibre by omics studies are often not related to the main structural components, while the highly expressed wool keratins and KAPs may not differ markedly between different groups of sheep or goats.

It is also important to note that the difference in wool and cashmere traits may not necessarily result from changes in protein or mRNA levels. Variation in protein sequences and the fibre structures that they form may also contribute to fibre variation. For example, non-synonymous SNPs, commonly found in wool keratin and KAP genes [107], can lead to structural changes rather than alterations in expression levels. These changes may not be detectable by omics studies, which typically rely on detecting quantitative changes.

While a transcriptomic-based approach can be useful, it requires supplementation with more targeted studies on the wool keratin and KAP genes themselves. Identification of the full set of wool keratin and KAP genes and characterisation of genetic variation in these genes should become a priority to better enable the transcriptomic analysis of wool and cashmere.

## 6. Future Research Directions and Challenges

### 6.1. The Ongoing Identification of Wool Protein Genes from Sheep and Goats

The function of wool keratin and KAP proteins suggests the importance of identifying and understanding the genes that encode these proteins for genetic improvement of wool and cashmere fibre traits. This makes the identification of these genes a priority for future study.

In sheep, the full set of the wool keratin genes is thought to have been identified, providing a foundation for comparative studies in goats, where research is more limited. Currently, only two wool keratin genes, *KRT31* [147] and *KRT33A* [148], have been investigated in goats. While this number is very small, the sheep orthologs could be used to facilitate the identification of corresponding genes in goats, making this task less challenging.

In contrast, a larger proportion of KAP genes remain unidentified in both sheep and goats, and their identification and characterisation will require effort. Determining whether any given gene is a *KRTAP* cannot be based solely on the amino acid composition of the protein it encodes (e.g., whether it is cysteine-rich or HGT-rich). As described above, the unique characteristics of KAP genes must also be considered, including their small size, their lack of introns, their chromosomal clustering, DNA sequence similarities with other known KAP genes, the presence of unique DNA sequence patterns, and their expression in skin and wool follicles. While other genes that have been called KAP genes are listed in some databases, their authenticity requires further validation. This might include those in KAP28-KAP35 families [149], whose sequences do not have many of the characteristics one might expect of KAPs.

Other challenges include using the correct nomenclature for these genes. This includes matching the genes to the best of our abilities to analogous human and other mammalian KAP genes yet accommodating species-specific differences and using universally accepted naming conventions, such as those promulgated by the HUGO Gene Nomenclature Committee.

In this respect, the matching of individual members of the sheep and goat KAP families to their human orthologs can be challenging, because for some KAP families, such as KAP1 and KAP6, the coding sequence of family members from one species are more closely related to each other than to the orthologs from other species. This is likely a consequence of species-specific concerted evolution [107,150].

It is also important that databases like Ensembl and GenBank are regularly updated, cross-referenced, and corrected to match recognised nomenclature, so that outdated gene names and incorrect sequences are identified, if not removed, and replaced with correctly identified sequences. Failure to do so means methods like GWAS and Ref-Seq, which rely on databases to identify genes, may perpetuate error rather than advancing understanding.

Continued efforts to identify wool protein genes ensure the use of recognised nomenclature and annotation, and the regular updating of sequence databases is essential for advancing our understanding of wool protein genetics. These efforts will provide a better foundation for investigating how genes affect wool and cashmere fibre traits, and they should be a major research focus.

### 6.2. Advancing Our Knowledge of Genetic Variation in Wool Protein Genes

Given the level of diversity in wool keratin and KAP genes, examining variation solely at the level of individual SNPs is insufficient. It is more appropriate to analyse genetic variation at the haplotype level, especially if this variation occurs in or near known genes. Accordingly, we believe extended haplotype analyses are required at the level of the clusters of wool keratin and KAP genes, especially if we are to obtain a comprehensive picture of genetic diversity, including variation within genes, between genes, and across groups of functionally related genes.

Haplotype analysis across the extended keratin and KAP gene clusters can also help identify potential copy number variation and chromosomal recombination events. In this respect, the KAP families typically consist of multiple members, with reports of up to 12 members for the KAP5 and KAP10 families in humans [124]. The high sequence similarity among gene family members suggests potential functional overlap or redundancy.

The question therefore arises as to why so many family members are needed to produce fibres. While bioinformatics analyses across mammalian genomes reveal varying numbers of KAP family members between species, intra-species variation in family size remains unexplored. Recombination within sheep *KRTAP* genes has been proposed, particularly within the KAP1 family [150], but broader investigation into recombination events across the keratin and KAP gene clusters, and the location of recombination hotspots is currently lacking. Addressing this will require accurate determination of extended haplotypes at the sequence level.

Given that each wool *KRT* or *KRTAP* has multiple alleles or variants, and no linkage of alleles or variants has been observed among the genes clustered on the chromosome, the potential number of haplotypes across the gene cluster is expected to be large. This would require the typing of a very large number of sheep to comprehensively screen and identify the potential haplotypes and obtain a thorough understanding of the genetic diversity present. This approach would require both high throughput and accurate extended haplotyping methods to be available.

Next-Generation Sequencing approaches enable higher-throughput sequencing by simultaneously sequencing multiple DNA fragments from a sequencing library. However, unless chromosomes are initially separated, the sequences obtained represent a mixture of maternal and paternal haplotype DNA, complicating the assembly of sequences to resolve haplotypes. Indeed, sequencing-based approaches often struggle to accurately define extended haplotypes, especially when sequence assembly is required and familial information is lacking. A whole-genome sequence study identified numerous poorly imputed regions across chromosomes, with the largest corresponding to the MHC region on OAR20 [151]. These findings highlight the complexity and challenges in sequencing and accurately assembling highly polymorphic regions, and in that respect, haplotyping of the KAP and wool keratin genes presents a challenge due to the high levels of polymorphism and the occurrence of gene duplications, repeat elements, and insertions and deletions, although they may not be as complex as MHC genes [152].

The recent development of phased long-range sequencing approaches can address these challenges by allowing DNA sequences from single-chromosome reads. These methods typically require pedigree information to identify haplotypes, but more recent approaches [153] describe chromosome-scale haplotype-resolved assembly of genomes. Long-range chromosomal haplotype sequencing approaches show promise, especially with Hi-C technology [154]. These methods could enable haplotyping of extended regions covering the KAP and wool keratin genes in individual sheep and goats, albeit possibly not at the throughput rate required for large-scale association studies.

A less challenging option is to examine haplotypes within shorter fragments or at the level of individual genes. Methods available for this include traditional clone and sequencing approaches, which enable the separation of individual longer DNA fragments (multi-kilobase fragments) for sequencing. However, this approach is time- and labour-intensive, making it unsuitable for high-throughput applications, such as those needed for large-scale association analyses to determine whether an extended haplotype affects a given wool trait.

Another approach used is gel separation-based analyses, such as the PCR-single strand conformation polymorphism (PCR-SSCP) technique. The PCR-SSCP approach uses gel electrophoresis to separate DNA strands of PCR amplicons and analyse sequence variation at the haplotype level, albeit in a defined amplified region. Initially regarded as suitable only for short PCR fragments (approximately 150 bp; [155]), PCR-SSCP has been demonstrated to be effective with a range of PCR product lengths up to approximately 640 bp [156,157,158]. For longer PCR products, a modified technique like polymerase chain reaction stem-loop conformational polymorphism (PCR-SLCP) can be utilised [159,160]. Gel separation-based approaches like PCR-SSCP are well suited for characterising the small KAP genes.

The use of PCR-SSCP analyses can also result in the detection of both known and hitherto unknown alleles and variants of genes. It enables the rapid exploration of genetic diversity on a large scale as the technique is simple and cost-effective, making it suitable for genotyping large numbers of samples [161,162]. Despite criticisms of its perceived simplicity and outdated nature, PCR-SSCP offers practical advantages over more sophisticated alternatives, including its lower set-up and run costs and easier implementation. The choice of method should prioritise meeting research needs and producing quality outcomes, regardless of perceived technological sophistication.

In sheep, 17 wool keratin genes have been identified, but genetic variation has only been examined in five type I genes (*KRT31* [74], *KRT33A* [71], *KRT34* [73], *KRT36* [65], and *KRT38* [65]) and four type II genes (*KRT81* [65,66], *KRT83* [64], *KRT84* [62], and *KRT85* [63,65]). Analyses to date have only covered fragments of these genes, leaving the full extent of genetic variation, especially in potentially regulatory regions, largely unexplored. No studies have addressed the remaining eight wool keratin genes. For goat wool keratin genes, genetic variation studies are sparse, limited to one gene, *KRT33A* [148].

Most KAP genes identified in sheep and goats have undergone analysis for nucleotide sequence variation, predominantly in coding regions, not upstream or downstream regions [107]. Future investigations need to explore non-coding regions and characterise variation in newly identified KAP genes.

Investigating genetic variation in wool protein genes through reliable haplotyping techniques will provide a comprehensive understanding of genetic diversity of wool genes and the complexity of genetic basis underlying wool and cashmere fibre traits. This information is essential for further research focusing on how these genes influence wool and cashmere traits and should be a key focus of future research studies.

### 6.3. Assessing the Effect of Wool Protein Genes and Validating Findings Across Breeds and Production Systems

Armed with knowledge of the genes, whether they are the ones described so far or those yet to be described, we can set about further testing how the genes and variation there-in affect the traits that determine the value of wool and cashmere fibre. This knowledge will be essential if we are to develop gene markers that might enable us to improve wool and cashmere quality and enhance production through strategic breeding efforts.

However, the associations reported to date are predominantly observed in only a few sheep and goat breeds and often involve limited sample sizes. It is therefore important to validate these associations across a much greater range of breeds and larger populations to ensure their robustness and reliability. This validation process is essential as it addresses the genetic diversity with different breeds and it can also potentially better explain the effects of specific environmental factors, nutritional influences, farm management practices, and other variables that affect fibre traits.

Ensuring the accuracy of fibre data measurements is also important. International standards for fibre testing are overseen by the International Wool Textile Organization (IWTO), and these standards ensure that fibre data measurements are consistent and comparable globally. While research may extend to other wool traits, emphasis should primarily remain on ascertaining the basis of key traits like MFD and variation therein.

Given the large number of wool keratin and KAP genes and their close clustering, unravelling the individual effect of each gene presents a major challenge. It will require the analysis of large numbers of genetically diverse sheep and goat breeds, for which robust phenotypic measures that are universally accepted are available. Rigorous statistical analyses will be required to minimise the effects of relatedness, environmental influences, error in phenotypic measurement, and sampling biases. While this will undeniably be demanding, they will be essential to advancing our understanding of how wool protein genes affect fibre traits and to facilitate the breeding of animals with more consistent fibre.

Once robust associations for individual KAP and wool keratin genes are established, their impact can be assessed, and different approaches for breeding to enhance specific traits can be developed. Given that these genes are clustered on chromosomes, closely linked gene variants are likely to be co-inherited by progeny unless recombination occurs. This linkage implies that desirable variants of some genes may be inherited alongside undesirable variants of other genes, and this will complicate selective breeding approaches. Accordingly, it may be necessary to select animals based on haplotypes encompassing multiple genes rather than selection based on individual genes. This approach ensures a more comprehensive consideration of the genetic makeup and its influence on fibre traits.

Future research is also needed to determine whether recombination occurs among KAP and wool keratin genes located on the same chromosome, and if so, the frequency and patterns, if any, of these recombination events. Understanding these recombination dynamics will enable the development of more effective breeding strategies. This information is important for developing selective breeding programs that will optimise specific traits, ultimately leading to enhanced production efficiency and product quality in the wool and cashmere industries.

Exploring the association of wool protein genes with fibre traits and validating these associations across diverse breeds will enhance our understanding of how these genes influence fibre characteristics. This research will facilitate the development of robust gene markers aimed at improving wool and cashmere fibre traits and should be a primary focus for future research.

### 6.4. Expanding Our Understanding of Wool Protein Gene Expression Patterns

Understanding the spatial and sequential expression patterns of wool and cashmere protein genes is crucial for unravelling their role in fibre development and characteristics. While some knowledge exists regarding these patterns in wool keratin genes in sheep, there is a dearth of information on goats. Moreover, the expression patterns of KAP genes remain largely unexplored, with data limited to a small subset identified decades ago in sheep.

Comprehending how genes are expressed within individual follicle cells is essential for understanding the intricate development of wool and cashmere fibres. These fibres are among the most complex protein structures found in mammals, characterised by a vast array of proteins assembled into a sophisticated structure. Each follicle cell contributes unique genetic and molecular cues that direct the assembly of fibres and influence fibre characteristics. However, pinpointing gene expression within these cells presents challenges due to the complexity arising from the dynamic genetic regulation, environmental factors, and cellular interactions that dictate fibre growth and composition. Advancements in molecular biology techniques, such as single-cell RNA sequencing and spatial transcriptomics, offer potential for revealing these complexities and understanding how gene expression profiles vary across follicle cells during different stages of growth and under varying conditions.

Real-time quantitative polymerase chain reaction (RT-qPCR) is a commonly used method for quantifying gene expression levels. However, challenges arise in using this approach for wool keratin and KAP genes due to their high sequence similarity. This can lead to cross-hybridisation of probes in in situ hybridisation assays and the amplification of non-target sequences in PCR-based approaches. Since designing qPCR primers that span two exons is not feasible for intronless KAP genes, primers may amplify both mRNA and genomic DNA indiscriminately, potentially leading to erroneous interpretation of gene expression levels.

To mitigate this issue, rigorous measures are required to ensure the removal of genomic DNA contamination from mRNA samples. While commercial mRNA extraction kits may eliminate most genomic DNA, trace amounts may persist. Employing a DNase treatment is a common strategy, but additional steps are necessary to validate the complete removal of genomic DNA.

One such approach involves conducting control experiments alongside RT-qPCR assays. By including a sample without reverse transcription in the reaction, it is possible to determine the presence of genomic DNA. The absence of an amplicon in this sample indicates successful genomic DNA removal, while the presence of an amplicon suggests residual DNA contamination. This careful experimental design ensures the reliability and accuracy of gene expression data, laying the foundation for comprehensive analysis of wool protein gene expression patterns.

Expanding our understanding of wool protein gene expression patterns is important for comprehending their roles in fibre development and characteristics. This research will elucidate how these genes influence wool and cashmere fibre traits and should be another focus for future studies.

Advancing the understanding of wool keratin and KAP genes, including genetic variation in them and their expression patterns, will provide a better picture of the complexity of wool protein genes and the genetic basis for the variability of wool and cashmere fibre traits. Integrating genetic diversity, expression profiles, and validated associations should enable the development of gene markers tailored for specific wool traits. These markers could then be employed in breeding programs to enhance wool quality and increase production efficiency, driving the advancement of the wool and cashmere industries.

## 7. Note on Terminology

In this paper, we use the term ‘wool keratins’ to refer to the orthologs of human hair keratins found in animals that produce finer or low-fibre-diameter fibres, such as those from sheep and cashmere goats. This term differentiates them from ‘hair keratins’, which refers to the keratins found in larger fibres in humans and animals. For consistency, we use the term ‘wool proteins’ to collectively refer to the proteins present in the smaller fibres, including those from sheep and cashmere goats.

## 8. Conclusions

Wool and cashmere fibres are compromised by natural variability in their properties that can impact their use and value. Despite progress in our knowledge of wool and cashmere proteins and genes, gaining a better understanding of their genetics and how it affects fibres remains a challenge, and more research is required to clarify gene and protein sequence variability and the location and patterns of gene expression. In this review, we have described future research directions and challenges, including the need for ongoing gene identification, variation characterisation, and gene expression analysis and association studies to enable further improvement to these valuable natural fibres.

## Figures and Tables

**Table 1:** Heritability estimates for selected wool traits.

Trait ^1^	Breed ^2^	Heritability ± SE ^3^	Reference
GFW	NZ Romney	0.35 ± 0.04	[3]
	Corriedale	0.52 ± 0.15	[4]
	Merino	0.24 ± 0.07	[5]
	Merino	0.29 ± 0.06	[6]
	Merino	0.46 ± 0.02	[7]
CFW	NZ Romney	0.36 ± 0.04	[3]
	Corriedale	0.37 ± 0.15	[4]
	Merino	0.31 ± 0.04	[8]
	Merino	0.28 ± 0.07	[5]
Yield	NZ Romney	0.40 ± 0.04	[3]
	Corriedale	0.75 ± 0.15	[4]
	Merino	0.58 ± 0.06	[5]
	Merino	0.35 ± 0.05	[6]
MFD	NZ Romney	0.57 ± 0.05	[3]
	Corriedale	0.65 ± 0.15	[4]
	NZ-DP	0.40 ± 0.10	[9]
	Merino	0.59 ± 0.06	[5]
	Merino	0.77 ± 0.02	[7]
	Merino	0.68 ± 0.01	[10]
FDSD	NZ-DP	0.27 ± 0.11	[9]
	Merino	0.51 ± 0.10	[11]
CVFD	NZ-DP	0.23 ± 0.10	[9]
	Merino	0.60 ± 0.06	[5]
	Merino	0.40 ± 0.02	[7]
	Merino	0.57 ± 0.02	[10]
MSS	NZ Romney	0.34 ± 0.14	[12]
	NZ Romney	0.24 ± 0.05	[3]
	Merino	0.39 ± 0.02	[11]
	Merino	0.13 ± 0.09	[5]
MSL	NZ Romney	0.41 ± 0.06	[3]
	Merino	0.71 ± 0.11	[5]
	Merino	0.48 ± 0.05	[8]
	Merino	0.54 ± 0.03	[13]
MFC	NZ-DP	0.31 ± 0.10	[9]

^1^ GFW—greasy fleece weight; CFW—clean fleece weight; Yield—wool yield; MFD—mean fibre diameter; FDSD—fibre diameter standard deviation; CVFD—coefficient of variation of fibre diameter; MSS—mean staple strength; MSL—mean staple length; MFC—mean fibre curvature. ^2^ NZ-DP: NZ dual-purpose sheep which are dominated by NZ Romney, Coopworth, Perendale, Texel and composite crosses of these breeds. ^3^ SE: standard error.

**Table 2:** Heritability estimates for selected cashmere fibre traits (fleece and down).

Trait ^1^	Heritability (±SE) ^2^	Reference
GDW	0.28	[14]
	0.30	[15]
	0.12 ± 0.12	[16]
CDW	0.62 ± 0.15	[17]
	0.61 ± 0.16	[18]
	0.61 ± 0.21	[19]
	0.57 ± 0.19	[20]
Yield	0.57 ± 0.15	[17]
	0.90 ± 0.23	[18]
	0.57 ± 0.20	[19]
	0.64 ± 0.21	[20]
MFD (whole fleece)	0.99 ± 0.19	[17]
	0.47 ± 0.15	[18]
	0.39 ± 0.16	[19]
	0.82 ± 0.23	[20]
SL (whole fleece)	0.29	[15]
	0.32	[21]

^1^ GDW—greasy down weight; CDW—clean down weight; Yield—down yield; MFD—mean fibre diameter; SL—staple length. ^2^ SE: standard error.

**Table 3:** Associations of ovine *KRTAPs* and *KRTs* with wool traits.

Gene ^1^	Sheep Type	Traits Associated ^2^	Ref.
**OAR1**			
*KRTAP7-1*	Kutta, Kari, Balkhi, Balkhi-cross, and Ramghani-cross	Yield, MSL	[35]
	Rambouillet	GFW, MSL	[36]
*KRTAP8-1*	Southdown × Merino	MFC, MSS	[37]
	Pakistani sheep	MSL, OpSD, CVOp	[38]
	Chinese Tan	CVFD in fine wool	[39]
	Chinese Merino	MFD	[40]
	Rambouillet	GFW, MFD	[41]
	Peppin Merino	MFD	[42]
*KRTAP8-2*	Chinese Tan	Fibre length, wool crimping	[43]
*KRTAP21-1*	Southdown × Merino	Yield	[44]
*KRTAP21-2*	Southdown × Merino	MSL	[45]
*KRTAP20-2*	Southdown × Merino	MFC	[46]
*KRTAP6-1*	Southdown × Merino	MFD, FDSD, CVFD, MFC	[47]
	NZ Romney	MFD, CVFD	[48]
	Chinese Tan	Fibre length, wool crimping	[49]
	Sandyno and Nilagiri	GFW, Yield, MFD	[50]
	Peppin Merino	MFD	[42]
*KRTAP22-1*	Southdown × Merino	Yield, MFC	[51]
	Barki, Rahmani and Ossimi	CR, SL, KS, GCG	[52]
*KRTAP6-3*	Southdown × Merino	MFD, FDSD, PF	[53]
*KRTAP20-1*	Southdown × Merino	GFW, Yield, MFD, FDSD, PF	[54]
	Chinese Tan	MFC in fine wool	[55]
*KRTAP36-1*	Southdown × Merino	PF	[56]
*KRTAP36-2*	Southdown × Merino	Yield	[57]
*KRTAP19-5*	Chinese Tan	MFC in fine wool	[58]
*KRTAP15-1*	Southdown × Merino	Yield, FDSD	[59]
*KRTAP26-1*	Southdown × Merino	MFD, FDSD, PF, MSL	[60]
*KRTAP28-1*	Southdown × Merino	MFD	[61]
**OAR3**			
*KRT84*	Gansu Alpine Fine-wool	MFD, CVFD, MFC, CF, MSL, MSS	[62]
*KRT85*	Southdown × Merino	GFW, CFW, PF	[63]
*KRT83*	Southdown × Merino	MFD, FDSD, CVFD, MFC, PF, Yield	[64]
*KRT86*	Chinese Merino (Xinjiang type)	MFD, crimp score	[65]
*KRT81*	Southdown × Merino	GFW, CFW	[66]
**OAR11**			
*KRT32*	Gansu Alpine Fine-wool	MFD, CF, MFC	[67]
*KRTAP3-2*	Rambouillet	GFW	[68]
*KRTAP1-1*	Merino and Merino-cross	FDSD, Yield at 24 months of age	[69]
*KRTAP1-2*	Southdown × Merino	GFW, CFW, Yield, FDSD, CVFD, PF, MFC, MSL, MSS	[70]
*KRTAP1-3*	Chinese Merino	MFD	[40]
*KRT33A*	Perendale	Fleece weight, Yield, MSL, MFC, crimp frequency, core bulk	[71]
	Merino and Merino-cross	FDSD, MSS	[69]
	Barki, Rahmani, Osseimi, Awase, and two crossbreds	CFW, MFD, MSL, MSS	[72]
*KRT34*	Southdown × Merino	MFD, FDSD, MSL	[73]
*KRT31*	Southdown × Merino	GFW, CFW, MSL	[74]
	Chinese Merino (Xinjiang type)	MFD	[65]
*KRT38*	Chinese Merino (Xinjiang type)	Crimp count	[65]
*KRT36*	Chinese Merino (Xinjiang type)	MFD, wool fineness	[65]

^1^ Genes are listed to reflect their order on the chromosome. ^2^ CF—comfort factor; CFW—clean fleece weight; CVFD—coefficient of variation of fibre diameter; CR—crimp percentage; CVOp—coefficient of variation of opacity; FDSD—fibre diameter standard deviation; GCG—greasy colour grade; GFW—grease fleece weight; KS—kemp score; MFC—mean fibre curvature; MFD—mean fibre diameter; MSL—mean staple length; MSS—mean staple strength; OpSD—standard deviation of opacity; PF—prickle factor; SL—staple length; Yield—wool yield.

**Table 4:** Associations of caprine *KRTAPs* and *KRTs* with wool traits.

Gene ^1^	Goat Type	Traits Associated ^2^	Ref.
**CHI1**			
*KRTAP8-1*	Inner Mongolia cashmere	Cashmere weight, cashmere length, hair length	[75]
*KRTAP20-2*	Longdong cashmere	Cashmere weight, MFD, cashmere length	[76]
*KRTAP22-2*	Longdong cashmere	MFD	[77]
*KRTAP6-5*	Longdong cashmere	MFD	[78]
*KRTAP6-2*	Longdong cashmere	MFD	[79]
*KRTAP20-1*	Longdong cashmere	Cashmere weight	[80]
*KRTAP15-1*	Longdong cashmere	MFD	[81]
	Jiangnan cashmere	CVFD	[82]
*KRTAP13-1*	Jiangnan cashmere	MFD	[82]
*KRTAP27-1*	Longdong cashmere	MFD	[83]
	Jiangnan cashmere	MFD, FDSD, CVFD	[82]
*KRTAP28-1*	Longdong cashmere	MFD	[84]
*KRTAP24-1*	Longdong cashmere	MFD	[85]
	Jiangnan cashmere	MFD	[82]
**CHI19**			
*KRTAP1-2*	Longdong cashmere	Cashmere weight	[86]
*KRTAP1-3*	Longdong cashmere	MFD	[87]

^1^ Genes are listed to reflect their order on the chromosomes. ^2^ MFD—mean fibre diameter; FDSD—fibre diameter standard deviation; CVFD—coefficient of variation of fibre diameter.

## Data Availability

Not applicable.
